# A Novel Tetrapeptide Ala-Phe-Phe-Pro (AFFP) Derived from Antarctic Krill Prevents Scopolamine-Induced Memory Disorder by Balancing Lipid Metabolism of Mice Hippocampus

**DOI:** 10.3390/nu16071019

**Published:** 2024-03-31

**Authors:** Jingqi Yang, Yan Qi, Beiwei Zhu, Songyi Lin

**Affiliations:** 1 SKL of Marine Food Processing & Safety Control, School of Food Science and Technology, Dalian Polytechnic University, Dalian 116034, China; yangjingqi523@163.com (J.Y.); qiyan202111@163.com (Y.Q.); 2 Engineering Research Center of Food, Dalian Polytechnic University, Dalian 116034, China; 3 National Engineering Research Center of Seafood, Dalian 116034, China; 4 Engineering Research Center of Special Dietary Food, The Education Department of Liaoning Province, Dalian 116034, China

**Keywords:** Antarctic krill peptide, neuroprotection, cognition, oxidative stress

## Abstract

Memory impairment is a serious problem with organismal aging and increased social pressure. The tetrapeptide Ala-Phe-Phe-Pro (AFFP) is a synthetic analogue of Antarctic krill derived from the memory-improving Antarctic krill peptide Ser-Ser-Asp-Ala-Phe-Phe-Pro-Phe-Arg (SSDAFFPFR) after digestion and absorption. The objective of this research was to assess the neuroprotective effects of AFFP by reducing oxidative stress and controlling lipid metabolism in the brains of mice with memory impairment caused by scopolamine. The 1H Nuclear magnetic resonance spectroscopy results showed that AFFP had three active hydrogen sites that could contribute to its antioxidant properties. The findings from in vivo tests demonstrated that AFFP greatly enhanced the mice’s behavioral performance in the passive avoidance, novel object recognition, and eight-arm maze experiments. AFFP reduced oxidative stress by enhancing superoxide dismutase activity and malondialdehyde levels in mice serum, thereby decreasing reactive oxygen species level in the mice hippocampus. In addition, AFFP increased the unsaturated lipid content to balance the unsaturated lipid level against the neurotoxicity of the mice hippocampus. Our findings suggest that AFFP emerges as a potential dietary intervention for the prevention of memory impairment disorders.

## 1. Introduction

Memory impairment is an intricate neurodevelopmental condition distinguished by a decrease in memory, disorder in memory, and loss of memory [1]. Memory impairment may be worsened by the effects of aging, illness, stress, genetic predisposition to risk, and the impact of oxidative stress caused by free radicals attacking the body [2]. In the last ten years, there has been a significant rise in the occurrence of memory loss. Furthermore, the growing prevalence of memory impairment among the elderly has led to a rise in the incidence of dementia, Parkinson’s, and Alzheimer’s disease [3]. Based on projections, the global population afflicted with memory loss and behavioral disorders is anticipated to increase from 36 million in 2010 to over 115 million by the year 2050 [4]. Additionally, the global population is projected to reach 9.7 billion by 2050 [5], up from 6.9 billion in 2010 (www.populationpyramid.net, accessed on 15 March 2024). This demographic trend suggests a rise in the prevalence of memory decline disorders from 0.52% to 1.19% of the total population by 2050. Hence, it is crucial to create functional food products and supplements that may serve as accessible interventions to improve cognitive function and alleviate memory deficits.

More evidence has demonstrated that bioactive peptides from food play a significant role in protecting against neurological damage and improving memory impairment, such as pine nut antioxidant peptide, sea cucumber peptide, walnut antioxidant peptide, etc. [6,7,8]. Those findings suggested that oxidative stress and apoptosis are the neuronal metabolic degeneration and death, which in turn cause irreversible cognitive damage [9]. In addition, neurodegenerative disorders are associated with oxidative stress-related harm. In this process, an excessive amount of free radicals attack lipids in cells and cause memory disorder [10]. The brain experiences disrupted signaling transmission and material exchange between cells due to the excessive production of reactive oxygen species (ROS) caused by oxidative stress, which is strongly associated with a decrease in unsaturated lipid levels [11]. Hence, the reduction in the level of unsaturated lipids is associated with both memory deterioration and the susceptibility to Alzheimer’s disease [12].

Scopolamine, a tropane alkaloid derived from Solanaceae plants, serves as a significant anticholinergic agent that exerts its effects on the parasympathetic nervous system [13]. It has been confirmed to exacerbate oxidative damage in the brain, disrupt hippocampal function, and impede memory formation and retention [14]. Studies have shown that scopolamine induces oxidative stress in a manner reminiscent of the aging process observed in mice, leading to the accumulation of free radicals within cells and subsequent osmotic stress and generation of reactive oxygen species. This series of events ultimately contributes to the progression of memory degeneration and impairment [15]. Hence, Scopolamine has been frequently utilized in research to investigate drug-induced memory impairment [7,16].

In our prior investigation, we discovered that a peptide called Ser-Ser-Asp-Ala-Phe-Phe-Pro-Phe-Arg (SSDAFFPFR), derived from Antarctic krill, safeguards PC12 cells against oxidative stress by controlling ROS level and suppressing the production of Bax and Caspase-3 [17]. Moreover, the administration of SSDAFFPFR through the mouth enhanced memory function in mice with impaired memory caused by scopolamine by decreasing malondialdehyde (MDA) levels in the hippocampus and stimulating the expression of CREB and BDNF [16]. Proverbially, the oral administration of bioactive peptide is easily affected by enzymolysis of gastrointestinal and absorption of intestine. A novel tetrapeptide, Ala-Phe-Phe-Pro (AFFP), was identified following in vitro digestion and absorption of the peptide Ser-Ser-Asp-Ala-Phe-Phe-Pro-Phe-Arg (SSDAFFPFR). The tetrapeptide AFFP is a synthetic analogue of a memory-enhancing peptide derived from Antarctic krill SSDAFFPFR. AFFP is probably the efficient fraction of neuroprotection in SSDAFFPFR. Nevertheless, it remains uncertain the extent to which AFFP enhances memory.

Currently, over 60 peptides have been approved by the FDA for medical use, including some derived from marine sources [18]. In the UK, fish protein peptides are used in functional foods to help with emotional stress [19]. Most marine peptides are still being studied in vitro, with limited human trials conducted [18]. Clinical investigations have shown that food-derived bioactive peptides may enhance cognitive function in humans, and the supplementation of whey peptides and lactone glandular peptides has demonstrated potential in mitigating cognitive and memory deficits [20,21,22]. The study of marine bioactive peptides presents a promising avenue for research due to the growing interest in functional foods aimed at preventing memory impairment. Antarctic krill peptides, sourced from the abundant and nutrient-rich marine species, hold potential application value in the food and nutrition industry.

This research examined the structural features of AFFP with regards to its antioxidant properties, as well as its in vivo absorption and distribution characteristics. An experiment using mice to induce memory impairment with scopolamine was carried out to evaluate how AFFP can prevent memory impairment and its mechanism. This was achieved through behavioral experiments, examination of the cholinergic system, oxidative stress system, and unsaturated lipid levels. The research offers a theoretical framework for enhancing memory using Antarctic krill peptide, establishing a groundwork for the future development of functional food products that prevent memory impairment disorders.

## 2. Materials and Methods

### 2.1. Materials and Reagents

Fluorescein isothiocyanate (FITC) was provided by Maclean’s Biochemical Technology Co., Ltd. (Shanghai, China). AFFP, a peptide derived from Antarctic krill, and FITC-AFFP, which was AFFP labeled with FITC fluorescence, were synthesized by Nanjing Source Peptide Biotechnology Co., Ltd. (Nanjing, China). Unless otherwise indicated, all other reagents were of analytical quality. Assay Kits were from Nanjing Jiancheng Bioengineering Institute (Nanjing, China).

### 2.2. Animals Treatment

Male C57BL/6 mice, 7 weeks old and weighing 21 ± 2 g, were acquired from Liaoning Changsheng Biotechnology Co., Ltd. (Benxi, China) with SPF-grade certification. A controlled environment on a 50 ± 15% humidity level and a light/dark cycle lasting for a duration of twelve hours at 25 ± 2 °C was provided for them. Approval for the research was granted by the Animal Ethics Committee at Dalian Polytechnic University, with the assigned Ethics numbers DLPU 2023064 on 4 July 2023.

#### 2.2.1. Animals’ Treatment in Behavior Experiment

**Figure 1:**
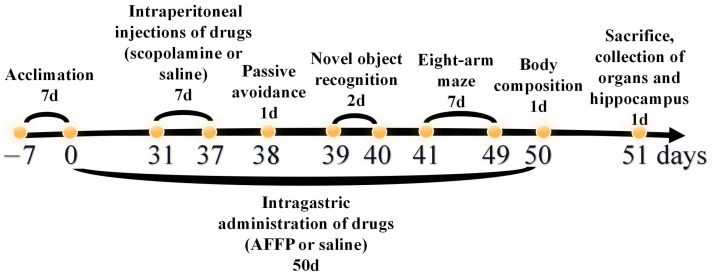
Schematic diagram of procedures for animal experiments.

The methodology for conducting animal experiments is outlined in Figure 1. The animal experiments were based on the research methodologies employed by Zheng et al. [16], Wu et al. [23], and Zhao et al. [24]. A total of twenty-seven male C57BL/6 mice of SPF grade were randomly distributed into three groups named control (administered with saline), scopolamine (administered with saline and scopolamine), and AFFP+scopolamine (administered with AFFP and scopolamine), with nine mice in each group. The weight of all mice was recorded every 7 days. The mice were acclimatized to the laboratory environment by being fed for a period of 7 days after their transfer from the supplier’s premises to the laboratory animal house, prior to the commencement of the experiment. Following a 7-day period of acclimation, during which the mice were provided only with normal food and water and not administered any drugs, they were subsequently orally administered drugs for a period of 50 consecutive days. On the 0–50th day, mice in the control group and in the scopolamine group were administered with 0.9% saline at 40 mg/kg via gavage, and mice in the AFFP+scopolamine group were administered with Antarctic krill peptide AFFP at 40 mg/kg via gavage. Additionally, on the 31–37th day, each group of mice received daily intraperitoneal injections of drugs (scopolamine and saline) two hours after receiving drugs (AFFP and saline) through gavage. Here are the details: mice in the control group were administered with 0.9% saline at a dosage of 1 mg/kg via intraperitoneal injection two hours after receiving 0.9% saline at 40 mg/kg via gavage, mice in the scopolamine group were administered with scopolamine at a dosage of 1 mg/kg via intraperitoneal injection two hours after receiving 0.9% saline at 40 mg/kg via gavage, and mice in the AFFP+scopolamine group were administered with scopolamine at a dosage of 1 mg/kg via intraperitoneal injection two hours after receiving AFFP at 40 mg/kg via gavage. The saline and peptide concentrations administered via gavage were 4.2 mg/mL, with a gavage volume of 0.2 mL, resulting in a total dose of 0.84 mg per mouse. The saline and scopolamine were intraperitoneally injected at a concentration of 0.105 mg/mL in a volume of 0.2 mL, totaling 0.021 mg per mouse. Both peptides and scopolamine were dissolved evenly in pure water at 25 °C before administration. The volume of drugs such as saline, AFFP, and scopolamine was based on prior research [16]. On the 38–49th day, behavioral experiments such as the passive avoidance experiment, novel object recognition experiment, and eight-arm maze experiment were conducted sequentially. On the 50th day, the measurement of the mice included the proportion of lean mass, fat, and water content. On the 51st day, the mice were euthanized via cervical dislocation following the extraction of blood from their eyeballs. Their organs such as brain, lung, liver, heart, spleen, kidney, jejunum, and hippocampus were collected and preserved for immediate experiments by being placed on ice, frozen in liquid nitrogen, and stored at −80 °C.

#### 2.2.2. Animals’ Treatment in Fluorescence Imaging Experiment

Another nine male C57BL/6 mice of SPF grade were randomly distributed into three groups named Control, FITC, and Fluorescein Isothiocyanate Labelled AFFP (FITC-AFFP), with three mice in each group. Prior to the experiment, the mice underwent a twelve-hour fasting period with unrestricted access to water. The FITC and FITC-AFFP+scopolamine groups were orally administered 10 mg/kg of FITC and FITC-AFFP, respectively, while the control group received 0.9% saline via gavage. Following a two-hour period of digestion, Mice were euthanized via cervical dislocation, and the brain, lung, liver, heart, spleen, kidney, and intestine were collected for the fluorescence imaging experiment.

### 2.3. Analysis of Nuclear Magnetic Resonance (NMR) Spectroscopy

The Bruker AVANCE III 400 MHz spectrometer (Rheinstetten, Germany) was utilized to acquire Nuclear Magnetic Resonance (NMR) Spectroscopy data, and the Deuterium exchange experiment, which identified active hydrogens, was conducted as described in a prior study [25]. A solution containing 10 mg of AFFP dissolved in 0.6 mL of Dimethyl Sulfoxide (DMSO) was prepared and transferred to an NMR tube for 1H NMR spectroscopy analysis. The 5 mm Broad Band Fluorine Observation (BBFO) probe was employed for detection, with a sampling delay of 6 s, a pulse width of 13 μs, and 32 recordings taken. Next, 90 microliters of Deuterium Oxide (D_2_O) were introduced to substitute the active hydrogen atoms within the peptide sequence. Following this, the same parameters were applied to identify the inactive hydrogen atoms in the peptide. Ultimately, all spectra were analyzed using Mestrenova 9.0 software.

### 2.4. Analysis of Absorption Distribution of AFFP In Vivo by Fluorescence Imaging

The distribution of fluorescence signals in the organs of mice subjected to the Section 2.2.2 “Animals’ Treatment in Fluorescence Imaging Experiment” were observed at a magnification of 4 × 10 by MIIS XFP-BIX fluorescence imager (Molecular Devices, CA, USA) and ECHO Revolve microscope (Semprex, CA, USA). The fluorescence intensity was measured by the Image J system, referring to the methods of Zhang et al. [26].

### 2.5. Passive Avoidance Experiment

The experiment was performed on the 38th day, following previous research conducted by a system (Chinese Academy of Medical Sciences, Beijing, China), which consisted of a grid floor, a light, and a dark compartment [27]. In detail, mice were placed inside a black box to exploit the natural aversion of mice towards light and their preference for darkness. Electric shock stimulation was utilized to facilitate the training of spatial learning memory in the mice. Consequently, mice would flee from the black box upon experiencing the electric shock. The frequency of each mouse re-entering the black box within a five-minute interval was meticulously measured by a stopwatch.

### 2.6. Novel Object Recognition Experiment

The experiment was conducted in a 40 × 40 × 40 cm cardboard box according to previous research [28]. On the 39th day, two identical red cylindrical blocks were placed symmetrically inside the cardboard box, after which the mice were free to move around for five minutes; the time of exploring two objects was recorded individually. On the 40th day, one block was replaced with a green square block; the time of exploring was recorded again. Mice that engaged in tactile or olfactory interactions with the objects at a distance of 2 cm or less were deemed to be exhibiting exploratory behavior towards the novel object. Neither direct climbing nor chewing on objects can be considered exploratory behaviors towards novel objects in mice, unless the mouse first sniffs the object it is climbing or chewing on [29,30]. These behaviors are more likely to be exhibited by mice that have become familiar with the objects. Objects were cleaned using 75% ethanol before each trial in order to eliminate any potential odor. The recognition index was displayed as the proportion of the total time of exploring the novel object to that of exploring both objects.

### 2.7. Eight-Arm Maze Experiment

During days 41–49, the experiment took place in an eight-arm maze and was analyzed using Smart 3.0 (Panlab, Barcelona, Spanish), with reference to the prior investigation [31]. The dimensions of each arm of the maze were 35 cm in length, 5 cm in width, and 25 cm in height. Prior to the examination, the mice body weight was accessed, and they underwent a fasting period of 24 h. Afterwards, the mice were given a consistent quantity of nourishment (2–3 g, modified according to their weight) each day in order to sustain their body weights at 80–85% of their usual consumption. The labyrinth was composed of eight branches, and nourishment pellets (4–5 pellets per branch, with a distance of around 3–4 mm between each) were scattered across the branches and central area of the labyrinth. After unlocking the doors to each arm, a total of four or five animals were positioned in the middle of the maze. Throughout the initial training phase spanning the 41–43rd day, mice were granted unrestricted access to both feeding and exploration within the maze for a duration of ten minutes in order to diminish their fear responses and facilitate their acclimation to an unfamiliar maze environment more efficiently. On the 44th day, they were trained separately. A food pellet was placed in every arm. After the pellets were ingested or following a duration of ten minutes, the mice were extracted from the maze. The identical process was repeated on the 45th day twice. On the 46–49th day, the pellet was only placed in the selected arms (arms 1, 2, 4, and 7). The entrances to these limbs were shut, and the rodents were positioned at the heart of the labyrinth. Following a brief 30-second pause, the entrances to the compartments were unlocked, granting the mice unrestricted mobility within the labyrinth and enabling them to consume the food pellets in each section. The experiment will be terminated if a pellet was not ingested within a time frame of ten minutes. Each day, there were three training sessions held, with breaks of over two hours in between. During the test, the measurement of working memory errors (WMEs) and reference memory errors (RMEs) took place. WMEs represent the frequency of mice re-entering the arm where they have already eaten food, indicating the presence of short-term memory. RMEs indicate long-term memory as mice enter the arm without food [32].

### 2.8. Body Composition Analysis

The Magnetic Resonance Imaging (MRI) analysis (MesoMR23-060V-1, Suzhou NIUMAG Analysis Instruments Corporation, Suzhou, China) was used to examine the distribution and composition of fat, lean mass, and water in the mice, following the previously described method [33]. The mice were given isoflurane to induce anesthesia before being placed inside the MRI machine for image capture. In one slice, a width of 3 mm was scanned for a 20 ms echo time and 500 ms repetition time. A 100 × 100 mm^2^ view was selected to scan a 3 mm wide slice with a 20 ms echo time and 500 ms repetition time.

### 2.9. Calculation of Organ Coefficients

Before being sacrificed, the mice underwent a 12-hour period of fasting, during which their weight was measured. The liver, heart, lung, spleen, and kidney were extracted, cleaned with saline solution, dried using dust-free paper, and weighed. The formula [34] was used to calculate the organ coefficients, which are expressed as a percentage of the organ weight divided by the mouse body weight multiplied by 100%.

### 2.10. H&E Staining in Liver, Spleen, Kidney, and Jejunum

Hematoxylin and Eosin (H&E) staining was conducted on all slices using the previously described method with certain alterations [35]. The microtome was used to slice all tissues into slices that were 4–5 μm thick. All tissue lesion locations were subsequently analyzed using a Nikon microscope (Eclipse E200-LED, Tokyo, Japan) at a magnification of 1000× under light microscopy.

### 2.11. Analysis of Acetylcholine (ACh) and Acetylcholinesterase (AChE) Levels

The hippocampus was removed and rapidly preserved in liquid nitrogen. The suspension was prepared by mixing it with normal saline and subsequently homogenizing it in a chilled water bath. The suspension was centrifuged at 8000× *g* for 10 min at 4 °C, and the liquid above was then collected [36]. The protein concentration was determined using the Bicinchoninic Acid (BCA) protein concentration assay kit. AChE activity and ACh content levels were measured using the acetylcholinesterase and acetylcholine assay kit, respectively.

### 2.12. Analysis of ROS Levels, SOD Activity, and MDA Level

The ROS probe dihydroethidium (DHE) fluorescence staining method was used to assess ROS levels, similar to previous methods [37]. DHE could easily enter living cells and fluoresce blue in the cytoplasm, and it was then oxidized by intracellular ROS including superoxide anion radicals to form ethidium oxide, which was incorporated into chromosomal DNA, causing the nucleus to fluoresce red. The intensity of red fluorescence production in cells allowed for the evaluation of ROS levels [37]. The frozen hippocampal tissue sections were thawed at room temperature and moisture levels were regulated. The tissue was delineated using a histochemical marker, followed by the introduction of a self-fluorescence suppressor for a 5-minute duration, and subsequent cleansing with water for 10 min. The DHE dye solution was then mixed with the delineated area and incubated in a dark incubator at 37 °C for 30 min. The slide was immersed in Phosphate Buffer Saline (PBS) at PH7.4 and subjected to three rounds of shaking on the decolorizing shaker, with each round lasting 5 min. The 4′,6-diamidino-2-phenylindole (DAPI) dye solution was added to counterstain cell nuclei and allowed to incubate at room temperature in the absence of light for a duration of 10 min. DAPI was a fluorescent dye with high affinity for DNA, commonly employed for the visualization of both live and fixed cells through blue staining. The slide was placed in PBS (PH 7.4) and moved around on the decolorizing table for three rounds, each lasting five minutes. Anti-fluorescence quenching sealing tablets were applied. Tissue section images were scanned by Pannoramic MIDI, Pannoramic 250FLASH, and Pannoramic DESK (3DHISTECH Ltd., Budapest, Hungary), and analyzed by CaseViewer 2.4 (3DHISTECH Ltd., Budapest, Hungary). Images were acquired by DAPI and Cyanine 3 channel. The DAPI excitation wavelength was 330–380 nm and the emission wavelength was 420 nm. The Cyanine 3 excitation wavelength was 510–560 nm and the emission wavelength was 590 nm. The nuclei exhibited a blue fluorescence signal in the DAPI channel and a red fluorescence signal in the CY3 channel which indicated ROS content. The fluorescence intensity of the red area in the CA1, CA3, and DG regions of hippocampus was quantified by the Image J software 1.47v. Assay Kit was used to measure the activity of superoxide dismutase (SOD) and MDA levels. Mice blood samples were acquired by subjecting them to centrifugation at a speed of 3500 rpm/min for 10 min at a temperature of 4 °C [38]. The liquid above the sediment was gathered for the examination of SOD and MDA.

### 2.13. Analysis of Saturated and Unsaturated Lipid Contents

The saturated and unsaturated lipid contents in the hippocampus were measured by Fourier Transform Infrared (FTIR) microscopy referring to previous method [39]. The brains were quickly frozen using cryogenic techniques and stored at −80 °C before being sliced into 12 μm sections using a CM1950 Cryo-microtome from Leica, Germany. The slices were observed on microscopy slides. The FTIR data were obtained with a Spotlight 400 FTIR spectrometer from PerkinElmer in the United States. The data were collected in transmission mode within the range of 4000 to 800 cm^−1^. We used a detector made of MCT with a Focal Plane Array (FPA) of 128 × 128, which was cooled using liquid nitrogen. Spectrum IMAGE 1.8 (PerkinElmer, MA, USA) was used to analyze the FTIR spectra. Chemical mapping was recorded for specific absorption bands at 2955 cm^−1^ and 3012 cm^−1^. Furthermore, quantitative information was obtained by calculating the average intensities of 25 randomly selected pixels. The final lipid content was paralleled four times for each group of samples.

### 2.14. Statistical Analysis

The sample size was determined using prior experimental data reported in previous publications [16,23,24], without the use of a power calculation analysis. Non-parametric statistical methods were employed to mitigate the impact of the small sample size. The one-way analysis of variance (ANOVA) was performed, followed by Duncan’s post-hoc test, using SPSS version 19.0. Graphical data analysis was performed using Origin 8.5. The data were reported as the average plus or minus the standard deviation, and were deemed statistically significant when *p* < 0.05.

## 3. Results and Discussion

### 3.1. Evaluation of Active Hydrogen Atoms in AFFP

**Figure 2:**
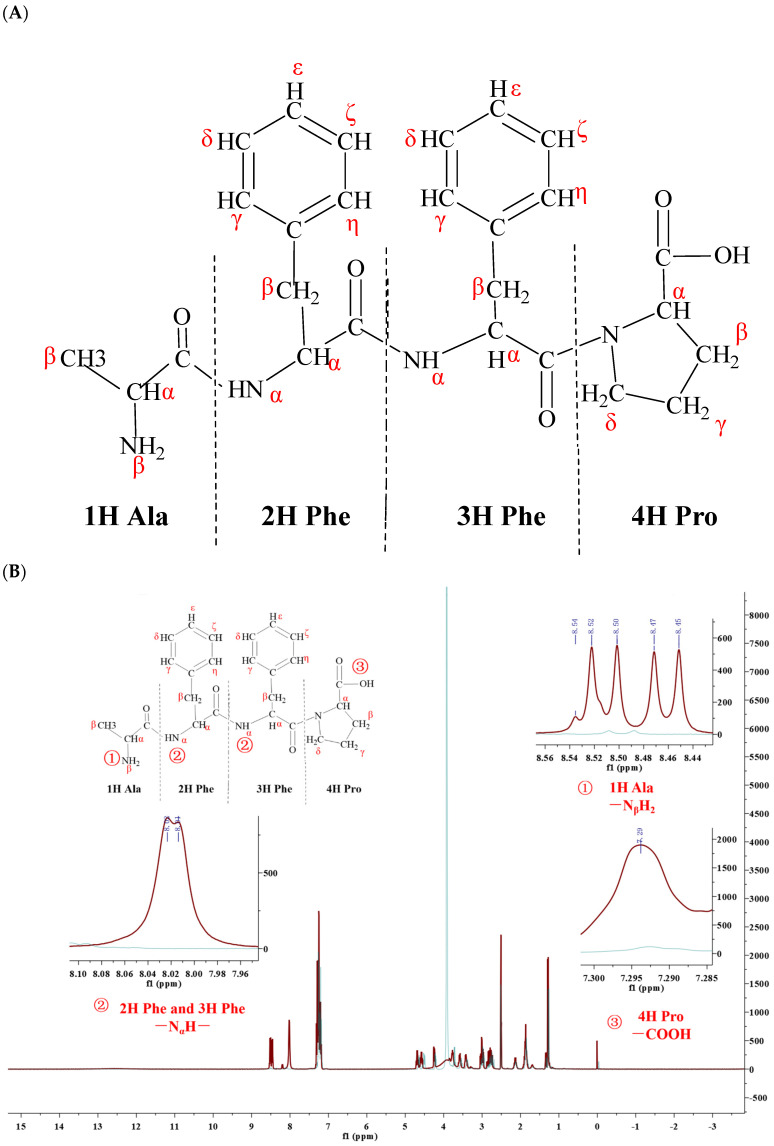
The MS/MS spectra and structural formula of AFFP. (**A**) Hydrogen proton coding diagram. (**B**) 1H NMR and active hydrogen chemical shift.

The potential bioactivity of the AFFP was ascertained by 1H NMR spectroscopy in terms of its ability to provide hydrogen protons. In organic chemistry, the carbon atom that was directly bonded to the functional group within a molecule was referred to as the α-carbon atom, while the hydrogen atom attached to this α-carbon was known as the α-hydrogen. Subsequent carbon atoms or hydrogen atoms were designated as β, γ, and so forth in a sequential manner. The graphical representation of the hydrogen protons within the AFFP is depicted in Figure 2A. The active hydrogen sites of the AFFP were identified by analyzing the differences in chemical shifts in the 1H NMR spectra before and after the addition of D_2_O. This is illustrated in Figure 2B. The active hydrogen sites of AFFP were found to be located at a chemical shift of 8.45–8.54 ppm, 8.01–8.02 ppm, and 7.29 ppm. The shift of 8.45–8.54 ppm was potentially indicative of the H protons on the -N_β_H_2_ group in 1H Ala, and the chemical shift of 8.01–8.02 ppm was observed for the H proton on the -N_α_H- group in 2H Phe and 3H Phe [40,41,42,43,44]. The chemical shift of 7.29 ppm was the H proton on the -COOH group of the 4H Pro [17]. It implied that AFFP potentially exhibited biological properties, such as antioxidant activity, owing to its spatial configuration, which offered the increased binding possibilities with free radicals attributed to the greater abundance of active hydrogen sites in its side chain.

### 3.2. Absorption Distribution of AFFP In Vivo

**Figure 3:**
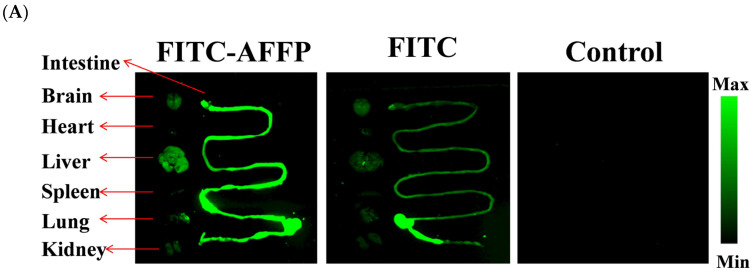

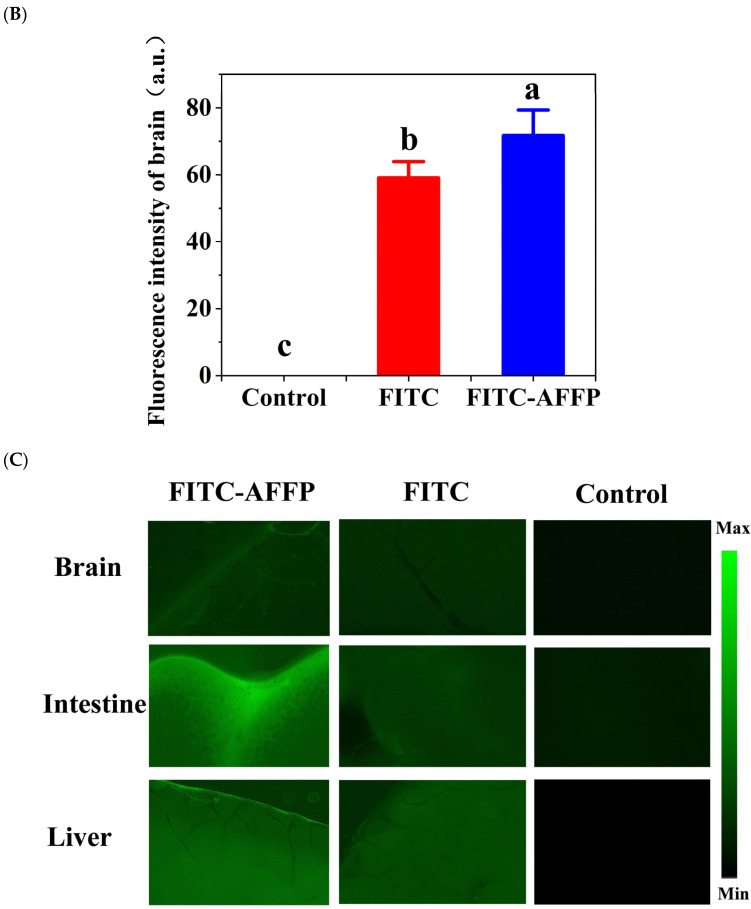
Absorption distribution of AFFP in vivo. (**A**) Fluorescence imaging of intestine, brain, heart, liver, spleen, lung, and kidney of mice by MIIS XFP-BIX fluorescence imager. (**B**) Fluorescence intensity in brain of mice. The different letters represent significant differences (*p* < 0.05). (**C**) Fluorescence imaging of brain, liver, and intestine of mice by ECHO Revolve microscope.

FITC was used as a fluorescent marker to conjugate with AFFP through the primary amine residue of Ala to evaluate the distribution of AFFP following oral dosing in mice. FITC could bind to the free amino group of peptides or proteins to form FITC-peptide/protein conjugates, thus making fluorescent peptides/proteins, which have been widely used to study the biodistribution of peptides in vivo [45]. The substance’s content and distribution in each organ were indicated by the fluorescent intensity [26]. Figure 3A shows there were no fluorescence signals in the control group, suggesting these organs had no autofluorescence. While mice displayed evident in vivo fluorescence signals after the administration of FITC and FITC-AFFP via gavage, the fluorescent signals were predominantly concentrated in the intestine, liver, and brain, with subsequent signals observed in lung and kidney. No apparent fluorescent signals were seen in the heart and spleen. In addition, Figure 3B illustrates the comparison of fluorescence intensity between the FITC-AFFP+scopolamine group and the FITC group. The fluorescence intensity of the FITC-AFFP+scopolamine group showed significant enhancements in comparison to the FITC group (*p* < 0.05). Figure 3C shows that a distinct fluorescent signal was seen in brain of the FITC-AFFP+scopolamine group compared to the FITC group. The blood–brain barrier (BBB) primarily consists of capillary endothelial cells with tight junctions, serving as a physical barrier to prevent the passage of proteins, drugs, or peptides into the central nervous system [46]. Despite this barrier, small peptides have demonstrated the ability to traverse the BBB, offering a potential advantage for targeting the central nervous system [47]. For example, the four amino acid peptide CAQK has been shown to successfully penetrate the BBB and selectively target demyelinating lesions, thereby potentially halting neurodegenerative processes and cognitive decline [48]. The findings of this study indicated that after digestion and absorption, AFFP was primarily distributed in the intestine, liver, and brain. However, due to potential degradation and metabolism by digestive enzymes in the gastrointestinal tract, it remained uncertain whether the resultant products were the original AFFP peptides or its metabolites. Further investigation is required to study the absorption and metabolism of AFFP in vivo using pharmacokinetic and metabolomics methods, as well as its metabolic pathway upon entry into the brain.

### 3.3. Assessment of Adverse Reactions of AFFP to Mice

**Figure 4:**
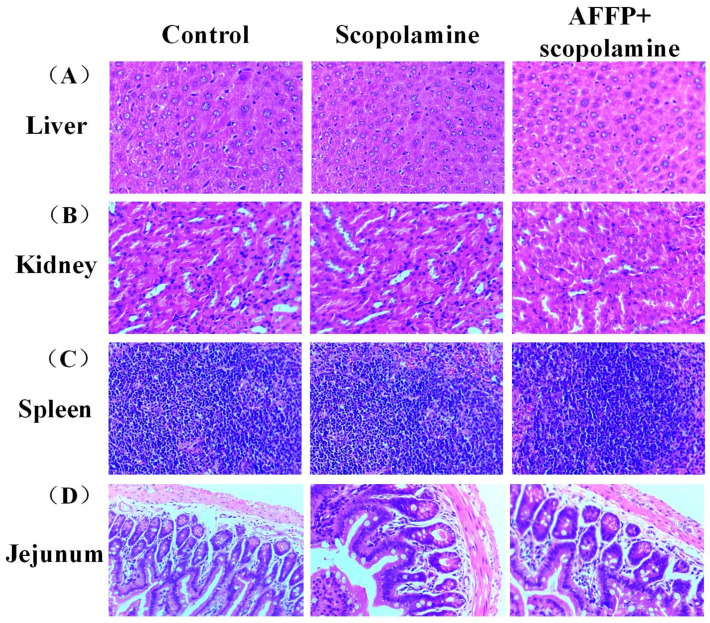
H&E staining of organs. (**A**) Liver. (**B**) Kidney. (**C**) Spleen. (**D**) Jejunum.

According to Table 1, the mice’s body weight exhibited a gradual increase over time, with no notable variation observed among the various groups (*p* > 0.05). According to the statistical findings, there were no notable variances in the proportions of lean meat, fat, and water in mice across the various groups (*p* > 0.05). At the same time, there were no notable variations observed in the organ sizes of the heart, liver, spleen, lung, kidney, brain, and hippocampus among the various groups (*p* > 0.05). No significant morphological alterations were observed in liver, spleen, kidney, and jejunum, as depicted in Figure 4A–D based on H&E staining. This was similar to the findings of Zheng et al. [16]. No notable variances in weight, composition, and organ ratios were observed among the various mouse groups following the administration of shrimp peptide via gavage [16]. The findings indicated that AFFP could be developed as a potential functional component without damage to organs of the body.

### 3.4. Effects of AFFP on Behavioral Experiments in Mice

**Figure 5:**
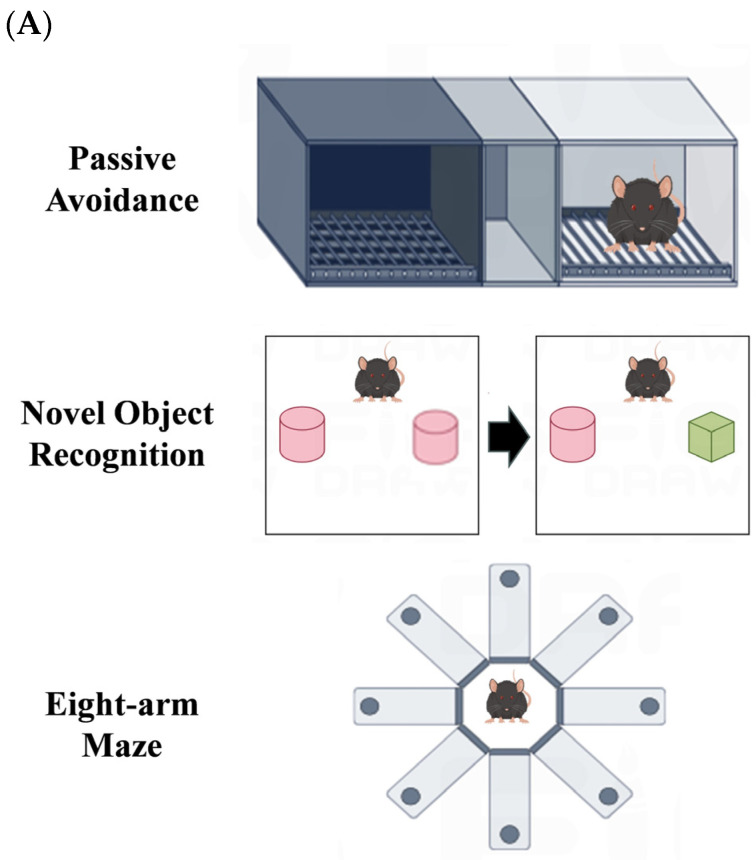

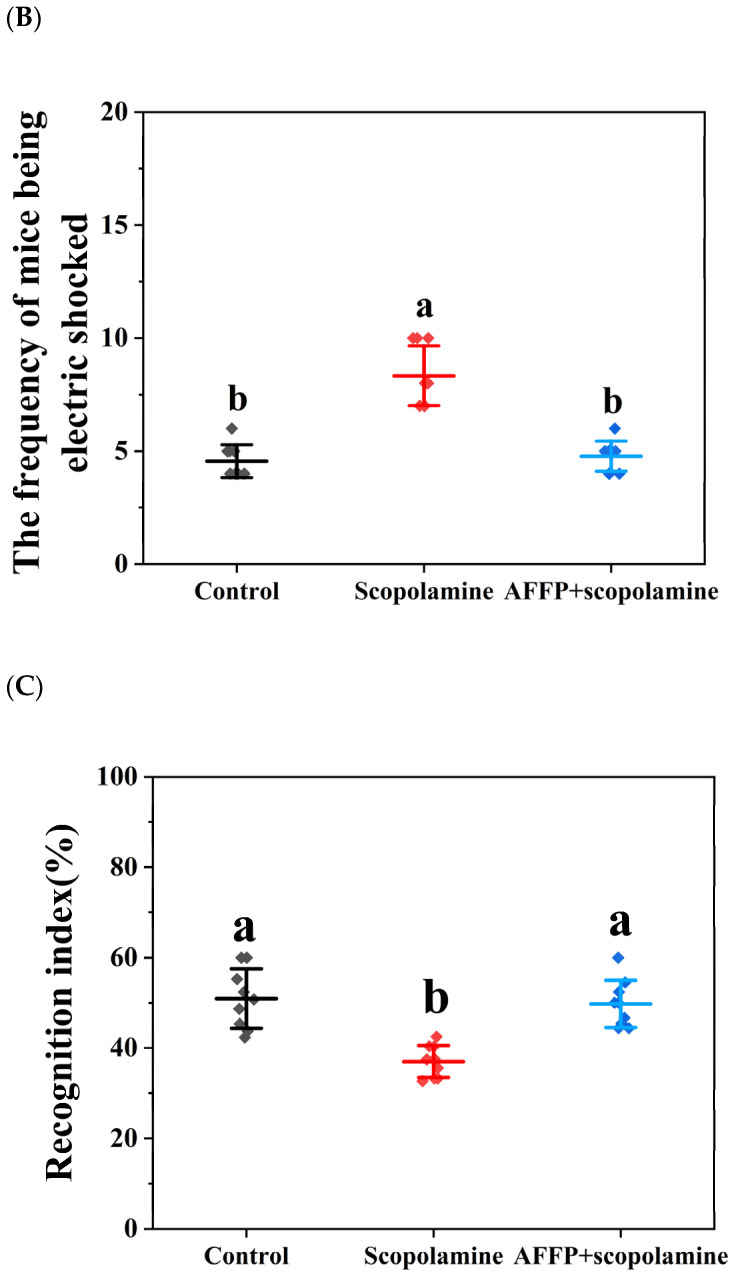

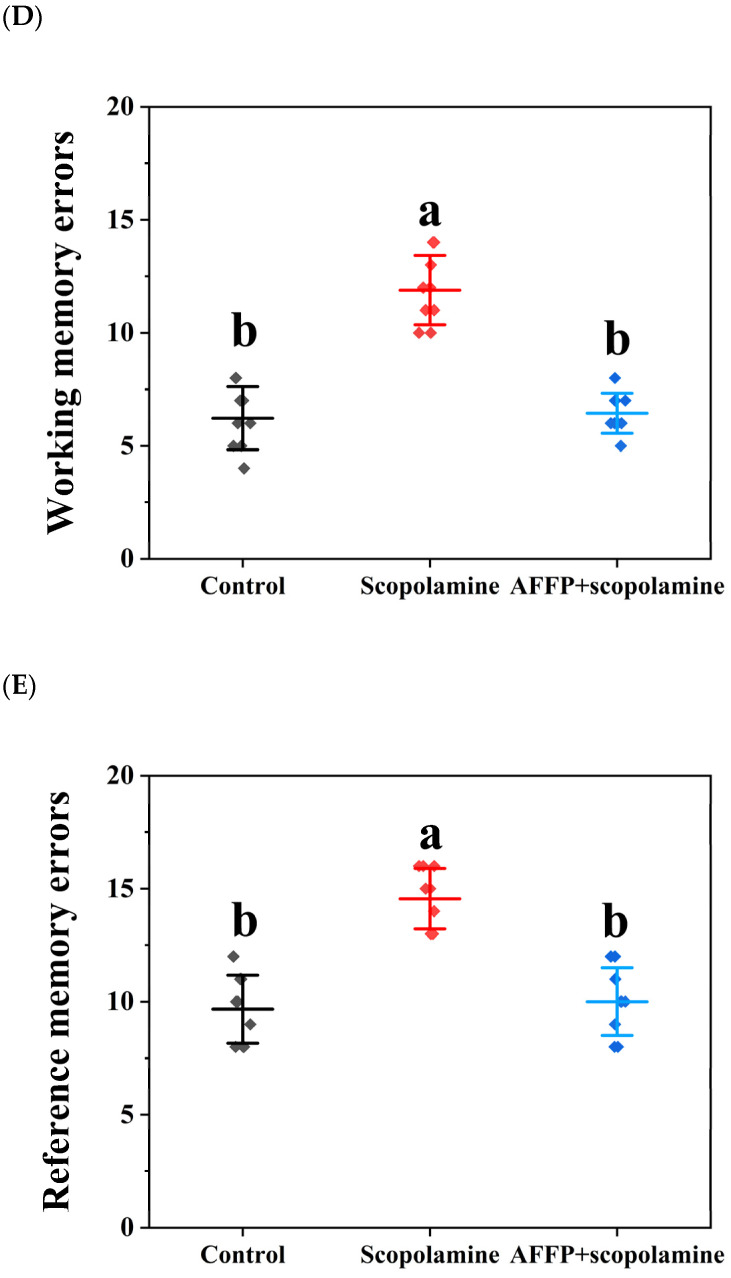
Results of behavioral experiments. (**A**) Experimental setup diagram of passive avoidance experiments, novel object recognition experiments, and eight-arm maze experiments. (**B**) Shocked time. (**C**) Discrimination index. (**D**) Working memory errors. (**E**) Reference memory errors. The different letters represent significant differences (*p* < 0.05).

Passive avoidance experiments, novel object recognition experiments, and eight-arm maze experiments are often used to assess the spatial learning and memory abilities of mice. During the passive avoidance tests (Figure 5B), the frequency of mice receiving an electric shock in the scopolamine group showed a significant increase compared to the control group (*p* < 0.05). Conversely, the AFFP+scopolamine group exhibited a significantly reduced avoidance in comparison to the scopolamine group (*p* < 0.05). According to Figure 5C, the mice in the scopolamine group had a noticeably reduced recognition index compared to the control group (*p* < 0.05). However, the mice in the AFFP+scopolamine group exhibited a notably higher recognition index than the scopolamine group (*p* < 0.05) during the novel object recognition experiments. Additionally, it was observed in Figure 5D,E that the scopolamine group showed a notable reduction in both working memory errors and reference memory errors compared to the control group (*p* < 0.05). Conversely, the AFFP+scopolamine group exhibited a notable rise in these errors in comparison to the scopolamine group (*p* < 0.05). Current studies [7] have shown a positive correlation between the shocked time in passive avoidance experiments and the severity of their memory disorders. Zhao et al. [24] discovered that mice given varying amounts of FYDWPK, ranging from low to high doses, exhibited enhanced memory performance in the passive avoidance test compared to the control group. A higher recognition index signified a heightened capacity for recognizing and remembering novel objects, while a lower recognition index indicated a diminished ability in this regard [49]. Our results were in line with the literature, in which SKF38393 HCl induced a long-term recognition memory enhancement by observing a higher value of discrimination index [50]. A higher level of learning and memory ability was suggested by the decreasing levels of working memory errors and reference memory errors [51]. The administration of 4′-hydroxyl-2-subsitiuted phenylnitronyl nitroxide (HPN) was discovered to have a significant and alleviating effect on the histological damages and spatial memory loss caused by hypobaric hypoxia (HH). This was supported by the reduced working memory errors and reference memory errors [52]. Overall, three types of behavioral tests conducted on mice indicated that AFFP alleviated the spatial differentiation and memory decline caused by scopolamine.

### 3.5. Effect of AFFP on AChE Activity and ACh Content in the Hippocampus of Scopolamine-Induced Mice

**Figure 6:**
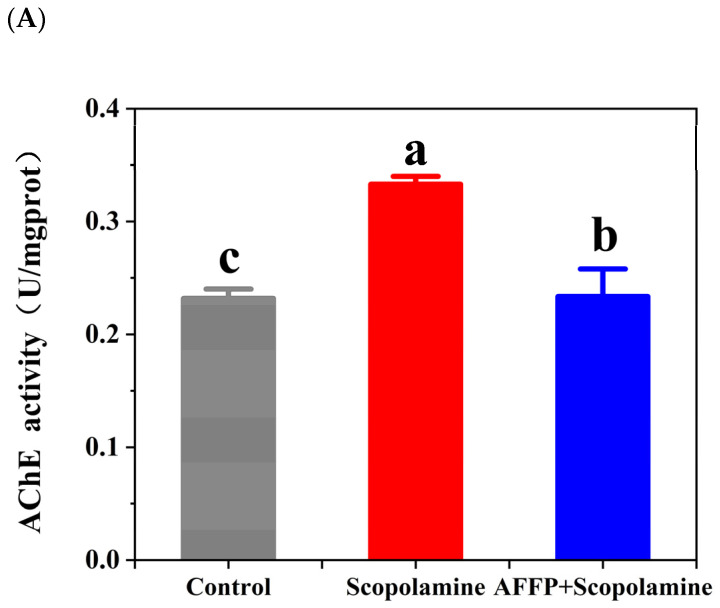

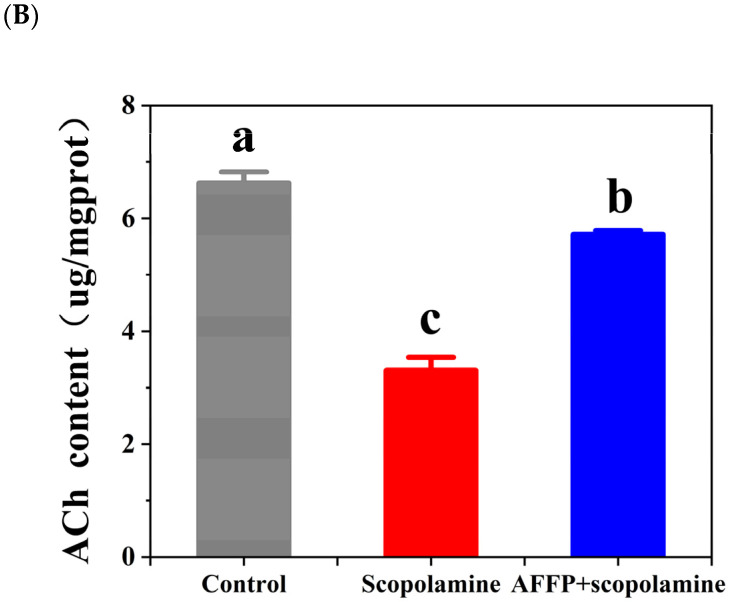
Effect of AFFP on the AChE activity and ACh content in the hippocampus of scopolamine-induced mice. (**A**) AChE activity. (**B**) ACh content. The different letters represent significant differences (*p* < 0.05).

Learning and memory are significantly influenced by the cholinergic system. Cholinergic biomarkers, namely AChE and ACh, were frequently employed to assess cognitive function related to learning and memory. The decrease in memory capacity is strongly linked to the disruption of the neurotransmitter system and alterations in hippocampal synapses [23]. Besides, overabundant breakdown of acetylcholine by acetylcholinesterase could hinder its typical attachment to receptors on the postsynaptic membrane, potentially interfering with the transfer of nerve signals between synapses and ultimately causing memory loss [53]. The activity of AChE could be enhanced by scopolamine, resulting in cognitive and memory impairments [54]. According to Figure 6A, the AChE activity in the scopolamine group significantly increased compared to the control group (*p* < 0.05). Nevertheless, AFFP significantly reduced AChE activity in comparison to the scopolamine group (*p* < 0.05). In addition, the Ach content in the AFFP+scopolamine group was unmistakably improved compared to that of mice injured by scopolamine (*p* < 0.05) (Figure 6B). The reason might be that AFFP acted on the active center of the AChE to inhibit enzyme activity. Similarly, Liu et al. [55] discovered that the activity and protein levels of AChE were notably reduced, while the levels of the neurotransmitter ACh were significantly elevated in the cerebral cortex of mice induced with scopolamine. This effect was observed after administering an extract and alkaloid fraction derived from Peganum harmala Linn. Wang et al. [8] found that hydrolysates from walnut protein could protect against memory loss by significantly increasing ACh levels and decreasing AChE activity (by 19.97–35.00%) in the hippocampus of mice treated with scopolamine. The findings of this study indicated that AFFP could potentially regulate the malfunctioning of the cholinergic system by enhancing ACh levers in the hippocampus and diminishing the AChE activity. While the mice administered with AFFP+scopolamine did not exhibit complete restoration to the baseline state of normal mice (without scopolamine injections), certain preventive and ameliorative effects were observed.

### 3.6. Effect of AFFP on Scopolamine-Induced Oxidative Stress

**Figure 7:**
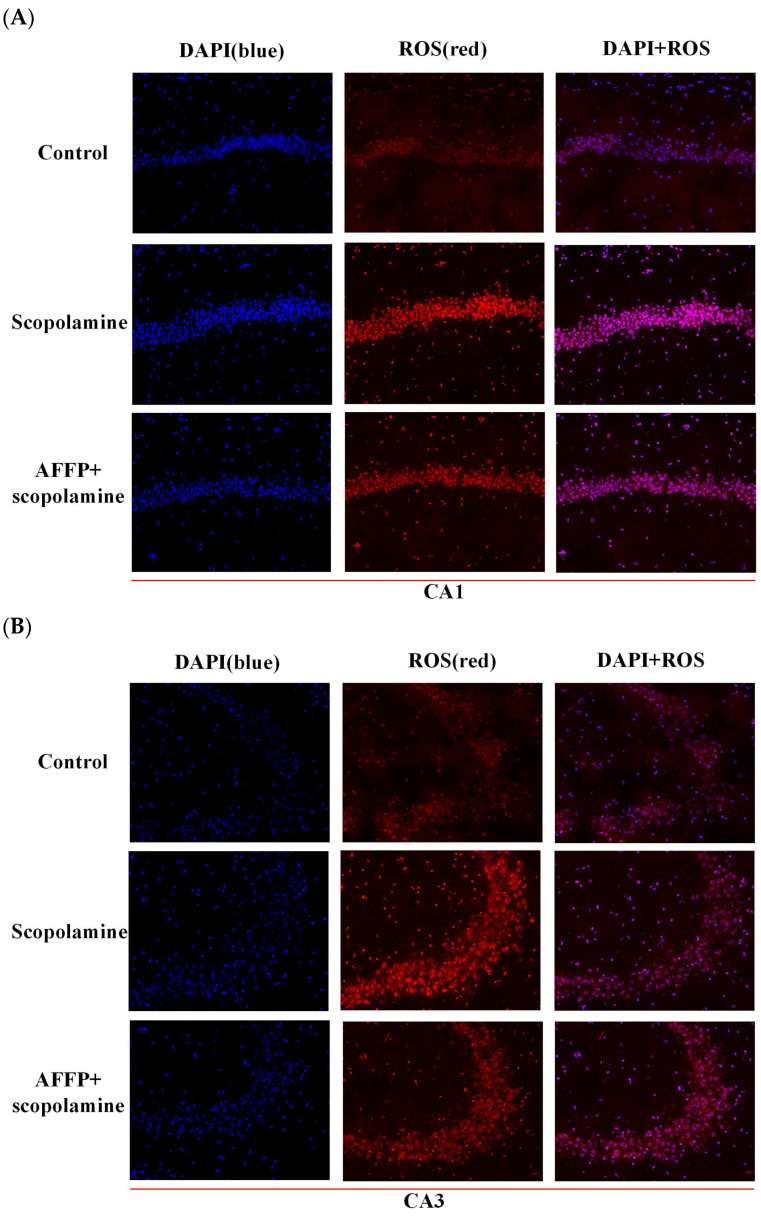

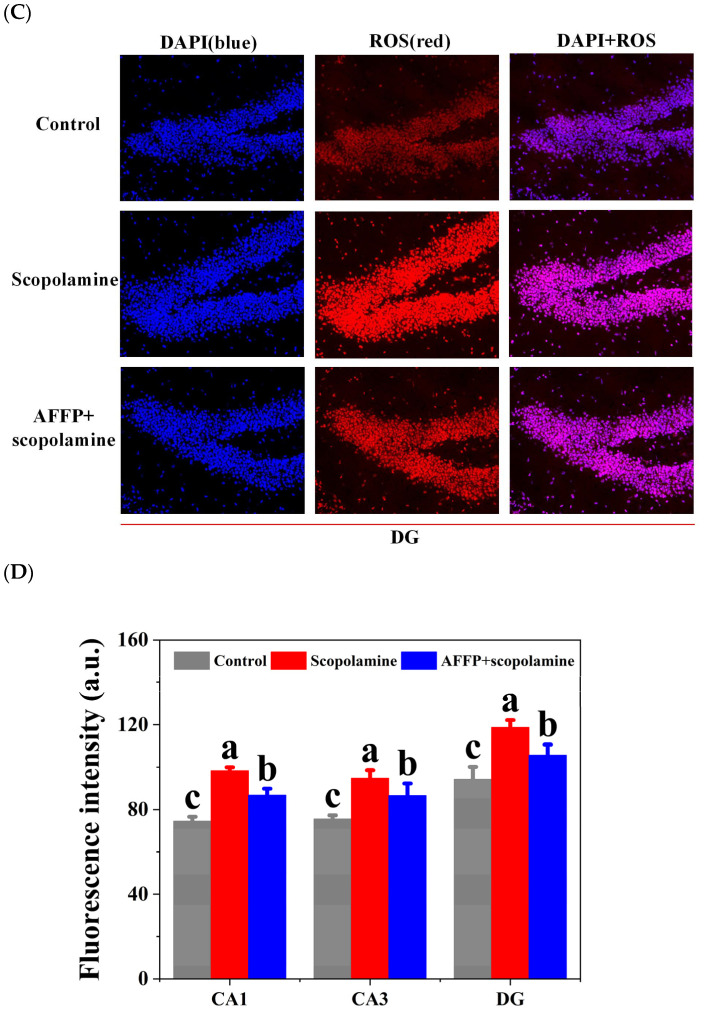

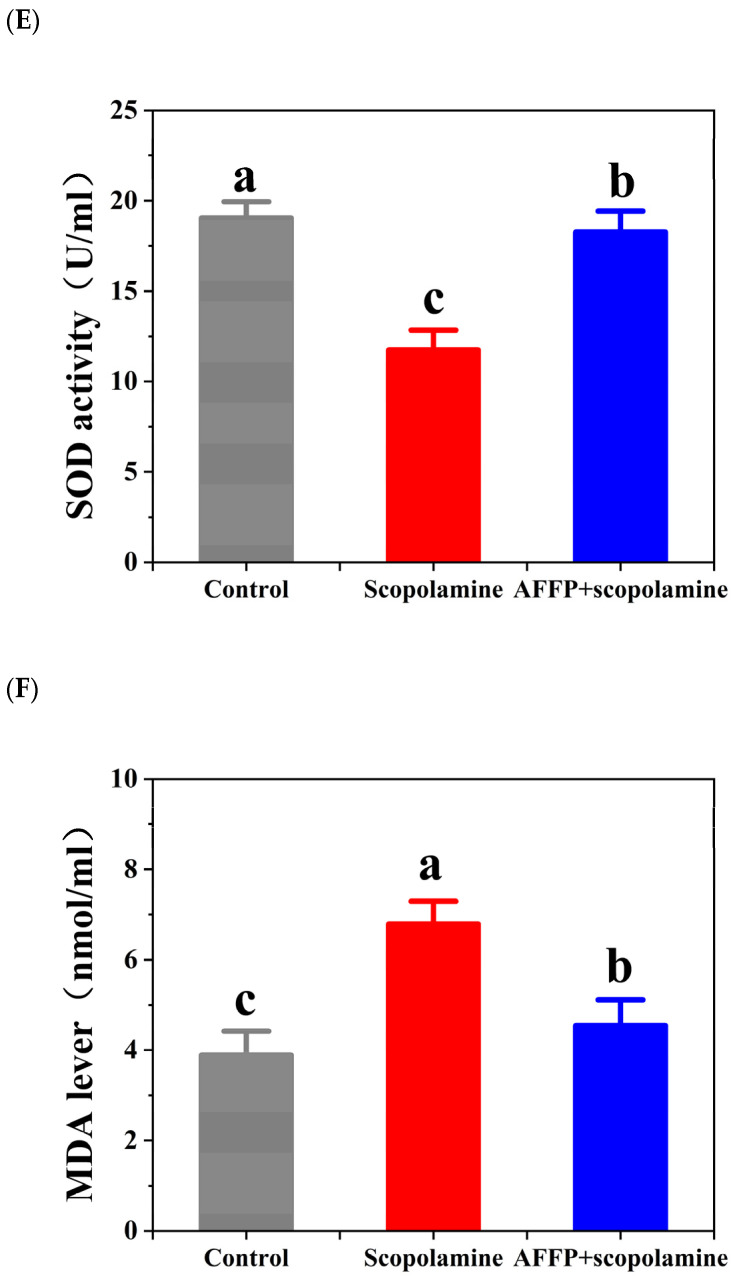
Effect of AFFP on scopolamine-induced oxidative stress. (**A**) Representative images of fluorescence staining in the CA1 region of the hippocampus. (**B**) Representative images of fluorescence staining in the CA3 region of the hippocampus. (**C**) Representative images of fluorescence staining in the DG region of the hippocampus. (**D**) ROS fluorescence intensity in the CA1, CA3, and DG regions of the hippocampus. (**E**) SOD activity in serum. (**F**) MDA levels in serum. The different letters represent significant differences (*p* < 0.05).

The administration of scopolamine may lead to an oxidative imbalance, resulting in neurodegeneration in the hippocampus and subsequently impairing memory function [23,54]. The hippocampus plays a significant role in cognition and memory. Various areas within the hippocampus play distinct roles in various forms of cognitive processes and memory. The CA3 region of the hippocampus potentially has a connection with the formation of long-term memories. On the other hand, CA1 might be associated with the process of discriminative learning. Additionally, the dentate gyrus (DG) is where information enters the hippocampus and is mainly made up of granule cells [56]. The peptides Met-Thr-Thr-Asp-Ile (MTTNI) and Met-Thr-Thr-Asp-Leu (MTTNL) purified from shrimp protein hydrolysate (SPH) have the potential to relieve immune dysfunction resulting from oxidative stress by increasing the activity of the intracellular antioxidant enzyme system [57]. In this study, the fluorescence intensity of the slice image was proportional to the amount of ROS. The accumulation of ROS was indicated by the significant increase (*p* < 0.05) in fluorescence intensity of various hippocampal regions (CA1, CA3, and DG) in the scopolamine group, as depicted in Figure 7A–D, when compared to the control group due to scopolamine induction. Nevertheless, prior application of AFFP significantly reduced the brightness when compared to the scopolamine group. Endogenous ROS caused astrocyte defects and neuronal dysfunction in the hippocampus to trigger impairment of cognitive function [58,59]. With the accumulation of excessive ROS in the hippocampus, MDA as a marker of lipid peroxidation increased, while SOD as a major scavenger of ROS was depleted [60]. According to the data presented in Figure 7E, there was a significant reduction in the SOD activity in the scopolamine group when compared to the control group (*p* < 0.05). The SOD activity was significantly higher in the AFFP+scopolamine group compared to the scopolamine group (*p* < 0.05). As shown in Figure 7F, the scopolamine group exhibited higher MDA levels compared to the control group (*p* < 0.05). The AFFP+scopolamine group showed a clear decrease in MDA levels compared to the scopolamine group (*p* < 0.05). Antarctic krill peptide AFFP effectively increased SOD activity and decreased MDA content compared to the scopolamine group. Our results were similar to Han et al. [61]. The researchers discovered that Schisanhenol boosted the SOD activity, reduced the MDA content, and effectively alleviated scopolamine-induced learning difficulties, thereby improving cognitive function. In summary, AFFP provided neuroprotection against cognitive decline caused by scopolamine by reducing oxidative stress in mice. While the mice administered with AFFP+scopolamine did not exhibit complete restoration to the baseline state of normal mice (without scopolamine injections), certain preventive and ameliorative effects were observed.

### 3.7. Effect of AFFP on Unsaturated Lipid Level of Hippocampus

**Figure 8:**
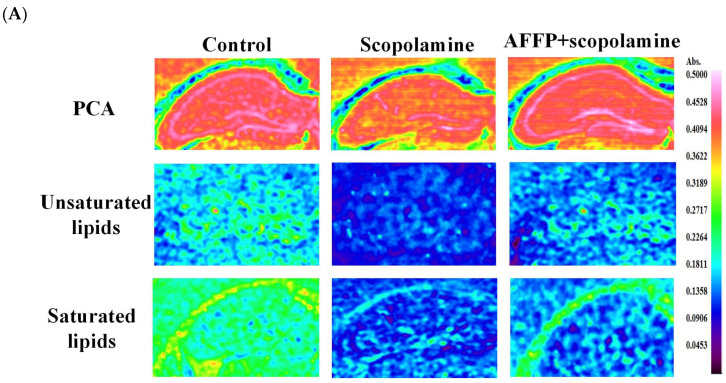

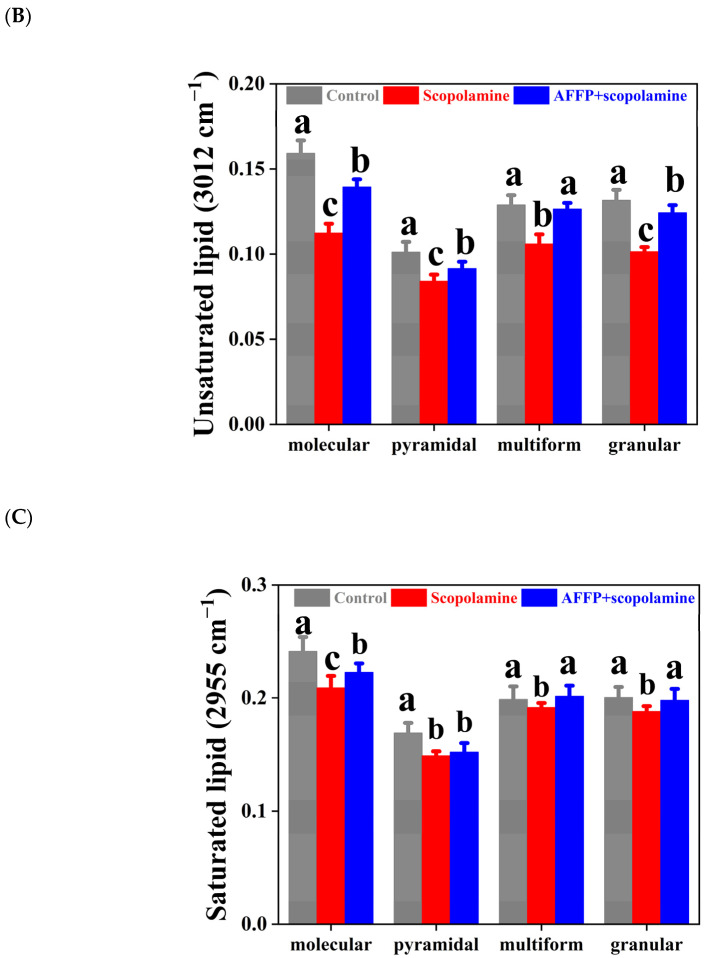

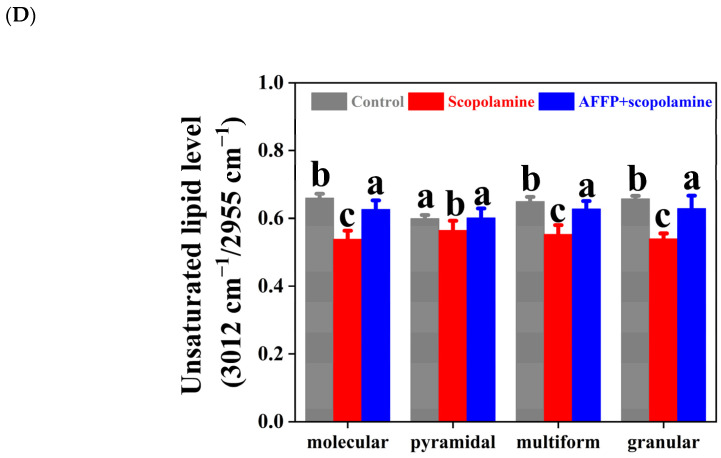
The unsaturated and saturated lipid content and ratio in the hippocampus of mice. (**A**) Representative chemical maps of selected absorption bands in the hippocampus. PCA was used to distinguish between various layers of the hippocampus, including molecular, pyramidal, multiform, and granular layers. (**B**) The unsaturated lipid content. (**C**) The saturated lipid content. (**D**) The ratio of unsaturated to saturated lipids. The different letters represent significant differences (*p* < 0.05).

The unsaturated lipid levers in the brain have a strong correlation with memory and neurological disorders, and they play a crucial part in the formation of the nervous system while also providing neuroprotective benefits for those experiencing memory impairment or the natural process of aging [62]. For a long time, it has been believed that polyunsaturated lipids were advantageous for cognitive function and memory. These lipids are recognized as regulators of neurotransmission and synaptic plasticity [63]. The levels of polyunsaturated lipids and saturated lipids impacted the cognitive function and memory retention of grown-up progeny [64]. Excessive ROS in the hippocampus would attack unsaturated lipid in biofilms and form lipid peroxides, triggering apoptosis and functional disorders of neurological systems [65]. Chwiej examined the dispersion of unsaturated lipid levels in the hippocampus by utilizing Fourier Transform Infrared (FTIR) microscopy. The absorption peaks at 2955 cm^−1^ (CH_3_ from lipids) and 3012 cm^−1^ (CH_2_ from lipids) were identified as the distinctive absorption signals for assessing the levels of saturated and unsaturated lipids [66,67]. According to the variances in cell composition [39], the hippocampus was categorized into four primary layers, namely, molecular, multiform, pyramidal, and granular layers. The study found that the hippocampus in the scopolamine group had significantly lower levels of unsaturated lipids and a lower percentage of relative saturated lipids compared to the control group. Conversely, the AFFP+scopolamine group exhibited notably elevated amounts and proportion of unsaturated lipids in comparison to the scopolamine group (*p* < 0.05) (Figure 8A–D). These findings aligned with the results reported by Lu et al. [7]. Sea Cucumber Peptides had the potential to mitigate the decrease in unsaturated lipid contents and unsaturated lipid ratio in the hippocampus of scopolamine-induced mice. SCP increased polyunsaturated fatty acid levels, including linolenic acid, arachidonic acid, docosahexaenoic acid, and decreased saturated fatty acid levels, including palmitic acid and stearic acid. Yu et al. [64] documented that elevated levels of saturated fats, like palmitic acid, and reduced levels of polyunsaturated fats, particularly docosahexaenoic acid and arachidonic acid, in the brains of C57BL/6j mice resulted in impaired spatial memory and learning capabilities. Ueda et al. [68] found that mice provided with a diet rich in polyunsaturated lipids exhibited increased levels of polyunsaturated lipids in their brains, improved memory, and enhanced lifespan. Eicosapentaenoic acid and docosahexaenoic acid from unsaturated lipids have been found to influence synaptic transmission by modulating α-nicotinic acetylcholine receptors and can inhibit loss of the dopamine system in PD, improving learning and memory function and exerting neuroprotective and nutritional effects [69,70]. In general, AFFP enhanced the restoration of scopolamine-induced memory decline in mice by augmenting the concentration and ratio of unsaturated lipids in the hippocampus. While the mice administered with AFFP+scopolamine did not exhibit complete restoration to the baseline state of normal mice (without scopolamine injections), certain preventive and ameliorative effects were observed. Meanwhile, further research might be necessary to identify the major saturated and unsaturated lipids in the brain, particularly those that were significantly impacted by scopolamine treatment and protected by AFFP, as well as to elucidate how these substances affect memory.

## 4. Conclusions

To summarize, the current research affirmed that the Antarctic krill peptide AFFP prevented memory deficits in the scopolamine-induced mice. Although not fully reverting to pre-scopolamine injection baseline levels, some degree of prevention and improvement was still evident. NMR hydrogen spectroscopy revealed multiple active hydrogen protons in the secondary structure of AFFP, which could be the source of its antioxidant properties and neuroprotection. Behavioral experiments demonstrated that AFFP was able to enhance the learning and memory ability. Additionally, we assessed the cholinergic mechanism, oxidative stress response, and unsaturated lipid content, and discovered that AFFP had the ability to reduce neuronal cell harm caused by oxidative stress associated with SOD activity and ROS levels. It also controlled the cholinergic mechanism by decreasing AChE activity and enhancing the level of unsaturated lipids. Overall, this study suggested that the Antarctic krill peptide AFFP could provide a new therapeutic solution and a dietary supplement source for neurological disorders. The Antarctic krill peptide, derived from a species abundant in marine environments and possessing significant nutritional value, exhibits potential utility in the food and nutrition industry. To elucidate the mechanisms underlying peptide activity at specific target sites, further in vivo studies are warranted. Specifically, investigations into the gastrointestinal digestibility and absorption, distribution, utilization, and optimal dosage of the peptide are essential. Such research endeavors will facilitate the advancement and utilization of the peptide in functional and nutritional food products in the future. However, further investigation is required to elucidate the effects of AFFP on levels of unsaturated and saturated lipids, its metabolic activity within the body, its ability to traverse the blood–brain barrier, and its influence on the microbiome–gut–brain axis. Given the potential for differential responses to drugs between male and female animals, it is imperative that future experimental designs should incorporate a balanced representation of both sexes in order to provide a comprehensive assessment of the drug’s effects on animals.

## Figures and Tables

**Table 1:** The body weight, body composition, and organ coefficient of mice.

Parameter	Control	Scopolamine	AFFP+scopolamine
**Body weight (g)**			
7th day	22.111 ± 0.7607 a	21.956 ± 0.9825 a	21.456 ± 1.0026 a
14th day	22.478 ± 0.7032 a	22.533 ± 1.0368 a	22.733 ± 1.2816 a
21th day	23.344 ± 0.8946 a	23.067 ± 1.0198 a	23.111 ± 1.6405 a
28th day	23.767 ± 0.9862 a	23.556 ± 1.6501 a	24.478 ± 1.4567 a
35th day	24.233 ± 0.9925 a	24.344 ± 1.7529 a	24.911 ± 1.412 a
42th day	23.978 ± 0.9909 a	24.278 ± 1.4948 a	24.089 ± 1.3383 a
49th day	25.133 ± 1.3105 a	24.756 ± 1.7422 a	24.511 ± 1.5576 a
**Body composition (g)**			
Heart	0.539 ± 0.058 a	0.591 ± 0.0232 a	0.557 ± 0.0668 a
Liver	3.530 ± 0.3332 a	3.717 ± 0.2355 a	3.442 ± 0.2225 a
Spleen	0.251 ± 0.0241 a	0.271 ± 0.0382 a	0.242 ± 0.0403 a
Lung	0.687 ± 0.1088 a	0.668 ± 0.0944 a	0.690 ± 0.0905 a
Kidney	1.198 ± 0.0901 a	1.142 ± 0.1775 a	1.092 ± 0.0935 a
Brain	1.770 ± 0.1639 a	1.715 ± 0.0825 a	1.784 ± 0.0915 a
Hippocampus	0.167 ± 0.0125 a	0.168 ± 0.0146 a	0.183 ± 0.0124 a
**Relative organ coefficient (%)**			
Lean mass	23.688 ± 6.9014 a	23.267 ± 4.668 a	27.906 ± 3.9113 a
Fat	3.801 ± 0.5677 a	3.651 ± 0.7943 a	3.991 ± 0.6571 a
Water	0.907 ± 0.461 a	0.900 ± 0.2375 a	0.812 ± 0.2778 a

Data are expressed as mean ± SD. The different letters represent significant differences (*p* < 0.05).

## Data Availability

The data shown in this study are contained within the article.

## Abbreviations

AFFP	Ala-Phe-Phe-Pro
SSDAFFPFR	Ser-Ser-Asp-Ala-Phe-Phe-Pro-Phe-Arg
ROS	reactive oxygen species
MDA	malondialdehyde
FITC	fluorescein isothiocyanate
NMR	nuclear magnetic resonance
DMSO	dimethyl sulfoxide
BBFO	broad band fluorine observation
D_2_O	deuterium oxide
FITC-AFFP	fluorescein isothiocyanate labelled AFFP
WMEs	working memory errors
RMEs	reference memory errors
MRI	magnetic resonance imaging
H&E	hematoxylin and eosin
ACh	acetylcholine
AChE	acetylcholinesterase
BCA	bicinchoninic acid
DHE	dihydroethidium
PBS	phosphate buffer saline
DAPI	4′,6-diamidino-2-phenylindole
SOD	superoxide dismutase
FTIR	fourier transform infrared
ANOVA	analysis of variance
BBB	blood–brain barrier
